# Look at This Swelling: Retroauricular Mass as Atypical Progression in Long-Survivor Endometrial Endometrioid Adenocarcinoma

**DOI:** 10.7759/cureus.33380

**Published:** 2023-01-05

**Authors:** Marco De Felice, Elena Tammaro, Mariagrazia Tammaro, Giacinto Turitto, Rodolfo Cangiano

**Affiliations:** 1 Department of Precision Medicine, Medical Oncology, Università degli Studi della Campania Luigi Vanvitelli, Naples, ITA; 2 Department of Clinical Oncology, ASL Caserta, P.O. Piedimonte Matese, Caserta, ITA; 3 Department of Public Health, Università degli Studi di Napoli Federico II, Naples, ITA; 4 Department of Child, Women, General, and Specialized Surgery, Università degli Studi della Campania Luigi Vanvitelli, Naples, ITA; 5 Department of Oncology, AORN Sant' Anna e San Sebastiano, Caserta, ITA

**Keywords:** atypical pattern of progression, long-term clinical outcomes, adjuvant radiation therapy, retroauricular mass, endometrioid endometrial carcinoma

## Abstract

Endometrial endometrioid adenocarcinoma is the most common histology in gynecological malignancies. Most women present loco-regional relapsing or peritoneal and liver involvement within three years from diagnosis. However long-survivor patients may be affected by atypical disease evolutions. Here we describe an extremely rare case of retroauricular metastasis in a patient affected by endometrial endometrioid adenocarcinoma, who had a total abdominal hysterectomy and bilateral salpingo-oophorectomy six years earlier and subsequent salvage surgery three years later for loco-regional relapsed disease.

## Introduction

Endometrial cancer is the most common gynecological malignancy with over 60000 new cases reported every year in the United States; endometrioid adenocarcinoma is the most frequent histology and it’s estrogen related [1]. Endometrial endometrioid adenocarcinoma has a favorable prognosis considering that most patients present with early-stage disease; the pattern of spreading is related to the grade of differentiation, that’s why well-differentiated tumors tend to be localized while poorly differentiated often present locoregional pelvic or para-aortic lymph nodes involvement, vagina or adnexa metastasis, and distant spreading, mostly in lung, liver, bones mainly vertebra and scalp [2]. Here we present a unique case of retroauricular progression in a long-survivor patient affected by endometrial endometrioid adenocarcinoma, emphasizing how such atypical progression patterns may be increasingly frequent in consideration to our ability to better chronicize disease.

## Case presentation

A 75-year-old woman was admitted to the Emergency Department of Sant’Anna and San Sebastiano Hospital, Caserta, for acute abdominal pain and persistent vaginal bleeding in September 2013. Through colposcopy a diagnosis of endometrial endometrioid adenocarcinoma was made, then total abdominal hysterectomy and bilateral salpingo-oophorectomy (TAHBSO) had been carried out, highlighting an International Federation of Gynecology and Obstetrics (FIGO) Stage I, G3, p53 mutated disease. Four cycles of three-weekly carboplatin area under the curve (AUC) 5 plus paclitaxel and adjuvant External-Beam Radiotherapy (EBRT) 48.6 Gy in 1.8 Gy fractions given five days a week for six weeks were then administered. Subsequent follow-up was negative until March 2017, when magnetic resonance imaging (MRI) of the abdomen and pelvis described an inhomogeneous thickening of the superior and postero-lateral wall of the bladder appearing to be indissociable from the stump of previous hysteroannessiectomy and non-cleavable from rectosigmoid junction. Due to the loco-regional recurrence of the disease, in absence of distant metastases, she was treated with anterior and posterior evisceration with bilateral percutaneous ureterostomy and definitive left colostomy, as R0 surgery.

Subsequently, in August 2017, hepatic MRI highlighted a nodular formation of about 2 cm of maximum diameter with peripheral ring enhancement, diffusion restriction, and hypointensity on the hepatobiliary phase labeled as secondarism, that's why standard first-line chemotherapy was begun with VI cycles of only carboplatin AUC 5 in consideration of the clinical conditions of the patient (performance status 1-2). The treatment has been completed in December 2017, obtaining a complete radiological liver response.

**Figure 1:**
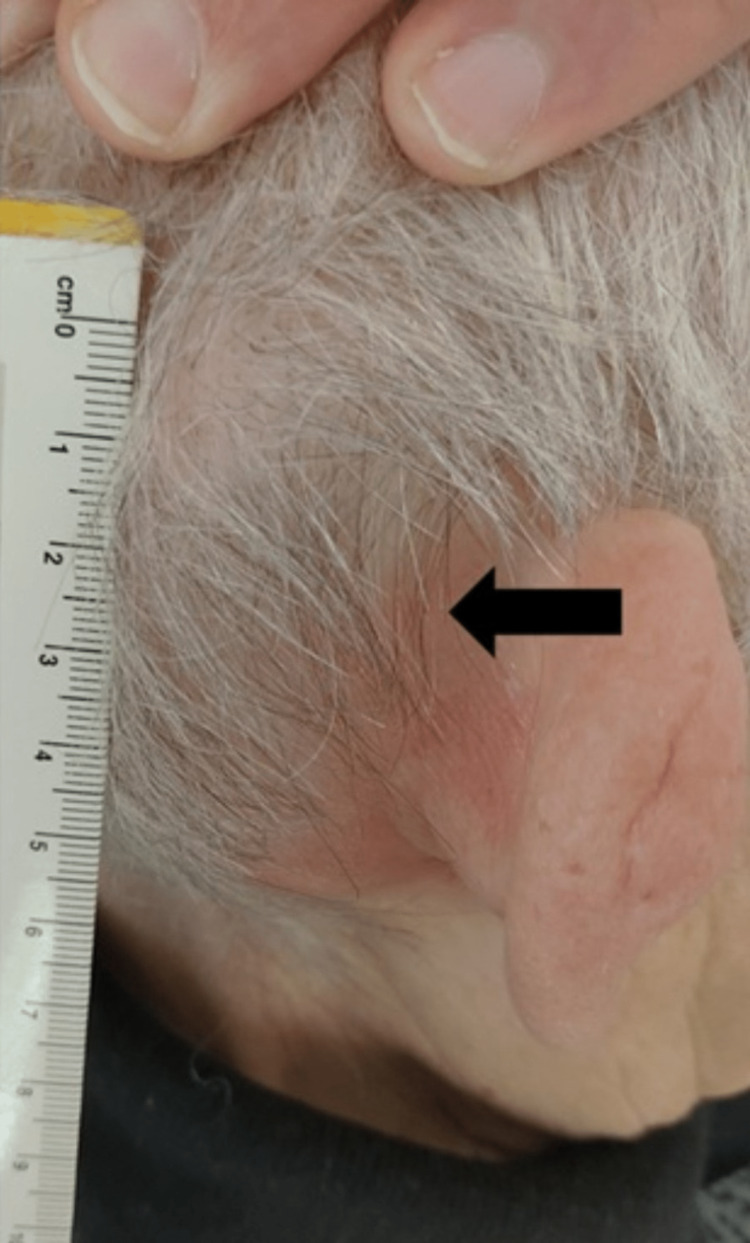
Retroauricolar swelling suspected for secondarism (black arrow)

**Figure 2:**
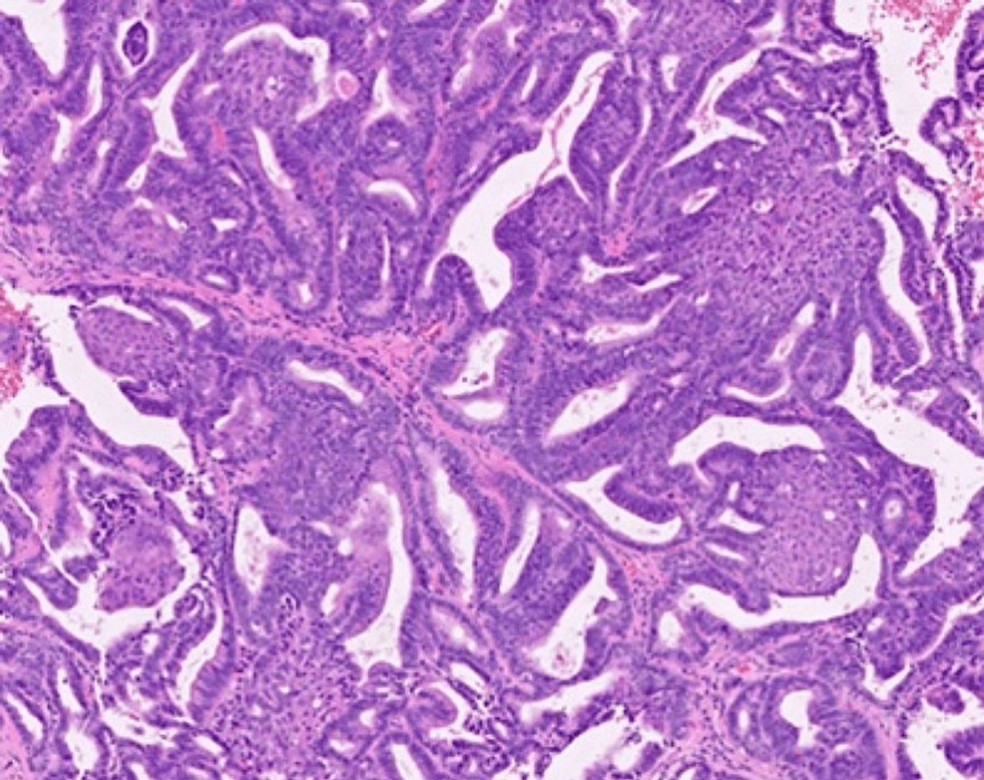
Biopsy diagnostic for metastasis from endometroid adenocarcinoma

**Figure 3:**
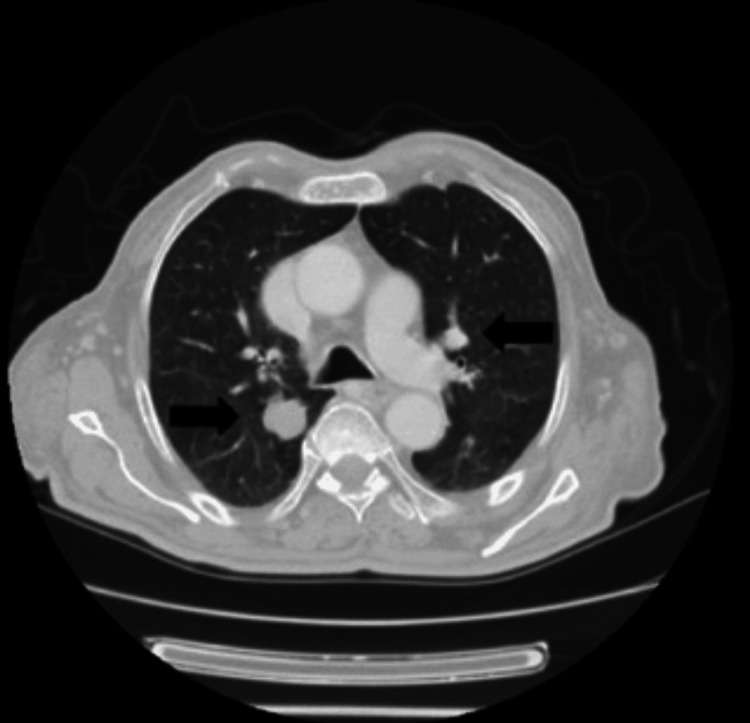
Lung metastases from endometrioid adenocarcinoma (black arrows)

**Figure 4:**
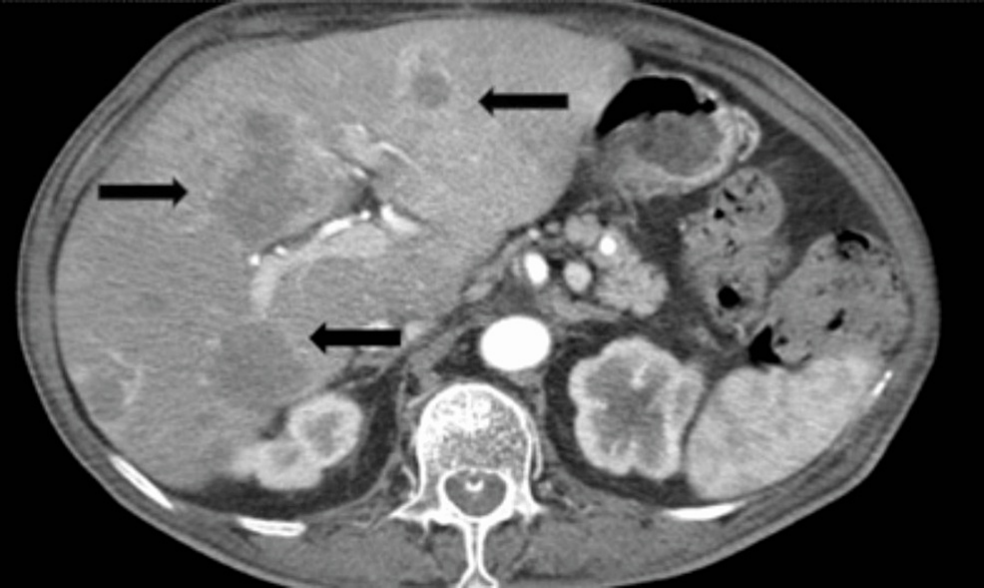
Liver metastases from endometrioid adenocarcinoma (black arrows)

During an outpatient visit in March 2019, an accurate physical examination revealed a right retroauricular swelling with hard consistency on palpation, fixed on superficial and deep planes, intact skin, retronuchal, and tempo-parietal spontaneous pain exacerbated by acupressure (Figure 1). Histological examination was compatible with metastasis of previous endometrioid adenocarcinoma (Figure 2). A restaging total-body computer tomography (CT) described osteolytic alteration of the right mastoid and part of ipsilateral occiput related to heteroplastic tissue of about 5 x 5 cm with calcifications, necrotic-colliquative areas and dyshomogeneous impregnation after contrast, laterally extending and infiltrating retro and supra auricular subcutaneous soft tissues, in contiguity with homolateral sigmoid sinus which appears to be thrombosed, indissociable from the posterior profile of the superior projection of the parotid gland; multiple bilateral pulmonary nodular lesions of 2 cm in greatest diameter (Figure 3), numerous liver metastases (Figure 4), the greatest of about 5 x 4 cm; osteolytic areas in left iliac wing, right ischiopubic branch, and right scapula. Positron emission tomography showed increased metabolic activity of the well-known right retroauricular tissue (SUV max 5.6), multiple lung lesions (SUV max 6.9), liver nodules (SUV max 6.7), concomitant occipital bone (SUV max 6.2), left iliac wing (SUV max 6.8), and right ischiopubic branch osteolytic lesions (SUV max 3.4).

**Figure 5:**
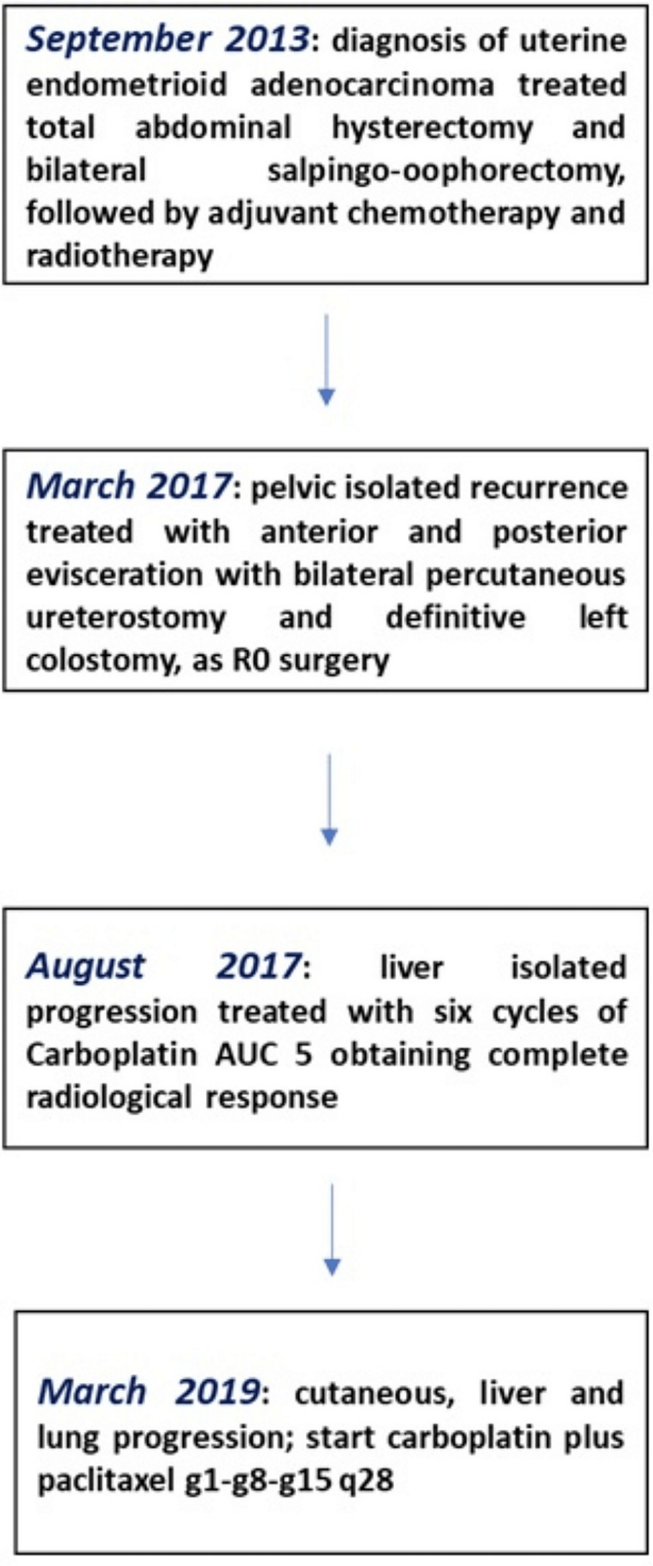
Clinical course of the disease

Considering the patient's clinical condition (performance status 2) and the extensive disease burden, second-line treatment was undertaken according to carboplatin AUC 2 plus weekly paclitaxel with schedule g1-g8-g15 q28 (Figure 5). After three months, retroauricular swelling was reduced, as well as the pain, with stable disease on liver and lung assessment. Unfortunately, after only five months of treatment, the patient died due to disease progression.

## Discussion

Endometrial tumors derive from epithelial cells of Mullerian origin and are divided into two variants with different pathogenesis: type I or estrogen-dependent endometrioid carcinoma and type II non-endometrioid carcinoma, non-estrogen dependent, represented by serous and clear cell carcinoma. Endometrioid adenocarcinoma has a favorable prognosis considering that the majority of patients present with early-stage disease [3]. The most common symptom at diagnosis is postmenopausal bleeding, followed by pelvic pain or pressure [3]. Surely surgical stage, or extent of tumor spread, represents the most relevant prognostic factor which is evaluated according to the FIGO staging system for endometrial carcinoma [4]. Metastasis typically occurred in late-stage disease including locally (pelvic or para-aortic lymph nodes, vagina, or bladder) and distantly, especially lung, liver, bones, or peritoneum [2,4]. However atypical pattern of recurrence may be reported: pancreas or periampullary secondarisms resulting in obstructive jaundice or pancreatitis have been recently described [5].

Cutaneous metastases may occur as the initial manifestation of various malignancies or during the course of the disease, with an incidence ranging between 0.7% and 10% [6]. Lung cancer (1.7 to 3.1%) and breast cancer (23.9%) are the commonest malignancies metastasizing to the skin in men and women respectively [6]. Even though endometrial carcinoma is the most common gynecological cancer, it rarely spreads to the skin, with a reported prevalence of 0.8%, potentially varying in number from a single nodule to more than 20 lesions [6]. Similar to cutaneous metastasis for other malignancies, the prognosis is poor, as widespread dissemination of the underlying malignancy is typically present [7,8]. Mean life expectancy is four to 12 months, even though survival was influenced by the time elapsed between diagnosis and the appearance of skin recurrences [7,8]. Clinically, cutaneous metastasis may be reported as nodules, papules, ulcers, and plaques, with usually four different histopathological patterns known as nodular, infiltrative, diffuse, and intravascular [9]. Treatment is primarily palliative, as chemotherapy and radiotherapy are largely ineffective, often denoting an extensive and aggressive disease [10]. In the case of isolated disease, electrochemotherapy may be proposed [10].

Being exceedingly rare, it may pose a diagnostic challenge. First of all correct strategy for cancer treatment and management is to determine histology, which is achievable with tissue biopsy: In our report, it enabled us to confirm a rare origin from endometrial endometrioid cancer. In fact, in previously published cases, cutaneous metastasis of endometrial cancer has been almost exclusively described at the site of initial surgery, that’s why surgical and radiotherapy areas must carefully be examined in order to exclude or confirm skin lesions [7]. In contrast, distant cutaneous sites, including the scalp, toes, and trunk, have been anecdotally reported [8,10]. Probably in our experience, the atypical pattern of cutaneous progression took place distantly since previously evisceration completely “sterilized” the area from oncological microscopical infiltration. Salvage cytoreductive surgery (SCR) has been shown to improve the survival of recurrent endometrial cancer, with a five-year overall survival rate ranging from 18 to 45 months, that’s why should always be proposed especially in high-volume centers [11,12].

Skin progression tends to be an indicator of aggressive disease, even if it typically occurs in long survivors [7,8]. Etoposide and cisplatin regimen was generally considered as efficacious as three-weekly carboplatin and paclitaxel combination in the treatment of advanced and recurrent metastatic endometrial cancer, as previously pointed out by Piver et al. [13]. However, in those years, the standard of care for women who experienced relapsed or progressive metastatic disease was an anthracycline, taxane, and platinum combination [14]. In our case, the choice of carboplatin AUC 2 plus weekly paclitaxel was carried out according to previous experiences of Secord et al. and Vandenput et al. reporting an overall response rate (ORR) ranging from 40% to 62%, especially in patients unfit for anthracycline and platinum combination due to poor performance status [15,16]. Nevertheless, disease evolution was rapidly fatal so the patient died five months after starting second-line treatment.

A new classification of the genomic landscape of endometrial cancer into four molecular subtypes with different prognoses, known as POLE, Mismatch Repair Deficient (dMMR), p53 abnormal, and No Specific Molecular Profile (NSMP) may be useful for numerous clinical trials exploring treatment escalation and de-escalation as well as matching targeted therapies to specific mutational or biomarker profiles, especially in the pattern of aggressive disease [17]. Surely our atypical experience has been influenced by p53 mutated status, historically denoting a high risk of recurrence and progressive disease even in FIGO stage 1 classification [17].

## Conclusions

Our report highlights how a better long-term disease control rate may lead to atypical progression patterns. Salvage surgery should always be proposed in high-volume centers for loco-regional isolated relapsed disease in order to improve overall survival, lowly impacting quality of life. Careful physical examination is essential to find significantly underestimated sites of disease. To date, skin metastases are extremely prognostically unfavorable especially if extra-pelvic, generally denoting diffuse visceral dissemination. p53 mutated disease is almost aggressive even in FIGO stage 1, however, actual molecular subclassifications may lead to the development of potentially practice-changing targeted therapies integrating therapeutic algorithms.
